# The Wanderings of Gut-Derived IgA Plasma Cells: Impact on Systemic Immune Responses

**DOI:** 10.3389/fimmu.2021.670290

**Published:** 2021-04-15

**Authors:** Selina J. Keppler, Marie Christine Goess, Julia M. Heinze

**Affiliations:** ^1^ School of Medicine, Institute for Clinical Chemistry and Pathobiochemistry, Technical University Munich, Munich, Germany; ^2^ TranslaTUM, Centre for Translational Cancer Research, Technical University Munich, Munich, Germany

**Keywords:** IgA antibodies, plasma cell (PC), inflammation, mucosal immunity, autoimmunity, gut-associated lymphoid tissues (GALT)

## Abstract

Humoral immunity is mainly mediated by a B cell population highly specialized to synthesize and secrete large quantities of antibodies – the antibody-secreting cells (ASC). In the gastrointestinal environment, a mixture of foreign antigens from the diet, commensal microbiota as well as occasional harmful pathogens lead to a constant differentiation of B cells into ASC. Due to this permanent immune response, more than 80% of mammalian ASC reside in the gut, of which most express immunoglobulin A (IgA). IgA antibodies contribute to intestinal homeostasis and can mediate protective immunity. Recent evidence points at a role for gut-derived ASC in modulating immune responses also outside of mucosal tissues. We here summarize recent evidence for wandering ASC, their antibodies and their involvement in systemic immune responses.

## Introduction

### Differentiation of Gut-Derived IgA Plasma Cells

Humoral immunity is mediated by a B cell population highly specialized to synthesize and secrete large quantities of antibodies – the antibody-secreting cells (ASC). ASC can be divided into proliferating plasmablasts (PB) and terminally differentiated, non-mitotic plasma cells (PC) (1). ASC represent a heterogeneous B cell subset that varies by location, secreted antibody isotype, and longevity. Most of our knowledge of ASC derives from the analysis of humoral immune responses towards immunisation and infection, which induce a transient germinal centre (GC) reaction and the differentiation of ASC with mainly an IgG isotype. The molecular requirements and signalling pathways involved in ASC differentiation, function and maintenance have been reviewed recently (2, 3). In the gastrointestinal environment, a mixture of foreign antigens from the diet, commensal microbiota as well as occasional harmful pathogens lead to a constant prevalence of GC and steady differentiation of B cells into ASC. Due to this permanent immune response, more than 80% of mammalian ASC reside in the gut (4). Most of these express immunoglobulin A (IgA), making IgA ASC an abundant B cell subset.

IgA contributes to intestinal homeostasis and can mediate protective immunity to enteric pathogens including viruses, bacteria, and fungi (5–7). The differentiation of B cells into IgA ASC mainly takes place in the gut-associated lymphoid tissues (GALT), including mesenteric lymph nodes (8), Peyer’s Patches (PP) (9) and isolated lymphoid follicles of the lamina propria (LP) (10, 11). Although debated, *in situ* LP IgA ASC differentiation has also been reported (12, 13). A variety of signals might modulate the differentiation of B cells into IgA ASC, dependent on the cellular composition, the soluble factors and location of the respective niche. Besides specialised dendritic cells, CD4 T follicular helper (TFH) cells (14), TH17 cells (15) as well as Innate Lymphoid Cells (12) have been implicated in the generation of IgA antibodies. In addition to stroma and other surrounding cells, these cells provide co-stimulation, as well as soluble factors to induce differentiation to IgA ASC, such as TGF-β (16), IL-21 (14), retinoic acid (17), and B cell activating factor (BAFF) or a proliferation-inducing ligand (APRIL) amongst others (18). However, the instructive sites, signals and cells involved are still not fully understood.

Precursors to IgA ASC include circulating naive follicular B2 cells, and innate-like peritoneal B1 cells (19–21). B cell differentiation in the GALT occurs *via* both T-independent (TI) and T-dependent (TD) pathways. The TI response to commensals mostly consists of polyreactive IgA antibodies with little somatic hypermutation or affinity maturation (22). TD responses require a GC reaction in which signals from TFH cells promote class-switch recombination (CSR) to IgA (14). Recent developments point at the fact that IgA secreting PC in the intestine are highly mutated in aged mice and humans (23, 24), which argues for a need of a TD response to generate sophisticated antibodies [reviewed in (25)].

Trafficking of IgA ASC is regulated by a combination of chemokine receptors as well as integrins on the surface of migrating cells. IgA ASC use α_4_β_7_ integrin to travel into the intestine. In addition, IgA ASC express the chemokine receptors CCR9, which is implicated in mediating entry into the small intestine (26) and all IgA ASC have been reported to express CCR10 (27). IgA ASC might use CXCR4 in order to travel to the bone marrow (28). Furthermore, homing to sites of inflammation might require the transient upregulation of CXCR3, as has been shown for IgG ASC (29).

Recent evidence points at a role for gut-derived ASC in modulating immune responses also outside of mucosal tissues, such as in the blood, the kidney, or the central nervous system (CNS). Here, we will sum up new developments on the field of wandering IgA ASC. We focus on “sightings” of IgA ASC, their contribution to systemic immune responses as well as open questions and possible future developments. We will exclusively focus on IgA ASC educated in the GALT and will not discuss IgA ASC educated in other mucosal tissues, e.g., the lung. We will use the term ASC to indicate PB and PC; also, if the exact distinction between those subsets is not clear from the literature.

## Involvement of Gut-Derived IgA ASC in Systemic Immune Responses

### Gut-Blood-Bone Marrow Axis

Evidence for the wandering of IgA ASC comes from studies describing IgA-secreting PB in the blood of healthy individuals (30). Those cells expressed CCR10 and α_4_β_7_ integrins and hence seemed to derive from mucosal immune responses. Similarly, monomeric IgA antibodies are readily detected in the sera of mice and men; but the source of these antibodies still remains to be defined in more detail. In patients suffering from celiac disease, clonal relatedness between gut PC and circulating serum IgA has been demonstrated using a proteomics approach combined with next-generation sequencing (31). These findings suggest that gut PC and serum IgA-producing PC derive from the same B cell clones. As IgA PC in the gut mainly produce dimeric IgA, the authors hypothesize that monomeric IgA is released by PC that migrated elsewhere. Interestingly, in human bone marrow (BM), approximately 5 – 40% of PC, as well as about 40 – 70% of PB express IgA as well as β_7_ integrin and CCR10, thus suggesting a substantial contribution of mucosal ASC to BM resident, long-lived ASC (30, 32). It might well be that these IgA ASC in the BM contribute to IgA levels in the serum of celiac disease patients.

Evidence for a gut-BM connection also stems from experiments in mice, in which homing of gut ASC to the BM has been demonstrated after oral immunization (33, 34). In mice, up to 70% of BM-resident PC are of the IgA isotype (35). While human individuals are constantly exposed to pathogens, it has to be considered that mice housed under SPF conditions display a more “naïve” immune system with little to no ongoing systemic immune responses. Hence, the main source of ASC is the constant immune response to the commensal microbiota in the GALT. Consequently, circulating IgA ASC can populate the PC niches in the BM with little competition from other PC, which might explain this substantial amount of IgA PC in the BM of mice. Interestingly, recent work demonstrates that a variety of commensal bacterial taxa induce TD IgA responses resulting in a marked increase in BM IgA ASC (36, 37). Wilmore et al. suggest that gut-derived IgA ASC in the BM secrete IgA antibodies into the blood circulation, thereby protecting against microbial sepsis induced by enteric pathogens (37). Which signals regulate the migration of gut-educated IgA PC to the BM niches as well as the potential triggers to release IgA into the serum remain to be determined.

### Gut-Kidney Axis – IgA ASC in Autoimmune Diseases

Circulating IgA antibodies are also involved in kidney malfunction in renal diseases such as IgA nephropathy (IgAN) or Systemic Lupus Erythematosus (SLE). IgAN is characterized by IgA immunocomplex deposition in the kidney mesangium (38). Increasing evidence points at a role of an aberrant immune response towards the intestinal microbiota (39) or dietary proteins (40) as cause for an exaggerated amount of systemic IgA. Interestingly, overexpression of the pro-survival factor BAFF in mice leads to increased levels of commensal-specific serum IgA and the development of IgAN dependent on the microbiome, thus supporting a direct link between the gut and the kidney (41). Again, the release of pathogenic IgA into the blood during IgAN might be related to BM ASC but has not been proven so far. Similarly, the migration of IgA ASC from the GALT to the BM or potentially also the kidney, as well as the eventual production of harmful IgA antibodies within the kidney during IgAN still remain to be elucidated.

Evidence for the presence of ASC in the kidney come from analysis of kidney biopsies from SLE patients, who often show glomerulonephritis induced by IgG deposits. This is likely due to aberrant B cell selection, activation and differentiation into auto-antibody producing ASC and correlates with increased serum levels of BAFF (42, 43). Interestingly, evidence from mouse models of SLE (44, 45) as well as patient samples (46) suggest the presence of IgG but also IgA-secreting PC in the kidney; concomitant with a higher abundance of these cells in the blood of SLE patients. Although present in kidney biopsy of SLE patients, IgA PC seem to be less frequent compared to IgG PC (47).

The prevalence of IgA-secreting PC in the kidneys of IgAN patients or in animal models of SLE remains elusive, but would be essential to study the homing to, the function in as well as composition of the kidney ASC niche during inflammation. If the infiltration of IgA ASC into inflamed organs further boosts disease through local antibody production remains to be investigated.

### Gut-Brain Axis – Protecting the Barriers

More and more findings recently highlight the role of IgA ASC in the central nervous system (CNS), in which IgA ASC play an unexpected yet essential role as a “brain firewall” to protect the blood-brain barrier (48). During homeostasis, mouse and human meninges – the membranes surrounding the brain and spinal cord – contain gut-derived and commensal-specific IgA ASC. These IgA ASC might contribute to an immunological barrier, thereby preventing the infiltration of pathogens into the CNS (36, 49).

During chronic inflammatory conditions such as multiple sclerosis (MS), commensal-reactive IgA ASC have been shown to play an immunoregulatory role during CNS inflammation in mouse and human. In a mouse model of MS, gut-derived, commensal-reactive IgA ASC can access the CNS and attenuate disease in an IL-10 dependent manner (36). In addition, elevating systemic IgA levels either through a commensal or overexpression of the ASC survival factor BAFF attenuates inflammation in the mouse model, suggesting a potential treatment option. Similarly, MS patients with active disease demonstrate an increased infiltration of commensal-specific IgA ASC in the cerebrospinal fluid (CSF), which could potentially be used as a marker for acute inflammation in MS (50). The anti-inflammatory capacities of these IgA ASC located in the CSF in MS patients are yet to be investigated. Furthermore, it remains to be speculated whether the meningeal-resident, gut-educated IgA ASC during homeostasis constitute the same population of IgA ASC found to infiltrate the CSF during inflammation in MS patients. Another open question is whether the meningeal IgA ASC are able to produce IL-10, or whether this population has maintained enough plasticity to acquire this phenotype as a response to dampen ongoing neuroinflammation.

### PC Survival Niches

Long-lived IgG-secreting PC derived from vaccination or natural infection with certain pathogens are known to persist for extended periods of time in the BM PC niche (51, 52). This niche is defined by a combination of cellular and molecular factors [e.g.: CXCL12, BAFF, APRIL, IL-6, integrins; reviewed in (53)] provided by stroma cells and a dynamic composition of immune cells (54). Recent evidence indicates that there might be more than just one type of niche for ASC, as BM stroma cells demonstrate a substantial heterogeneity (55, 56). It might well be that the ASC – stroma cell crosstalk shapes individual niches, which might be heavily influenced by the ASC themselves as well as the location of the niche (57).

Non-proliferating PC have also been found in brain biopsies of patients as well as in the meninges and the parenchyma of the inflamed spinal cord of mice during chronic inflammation in the CNS (58). These eventually long-lived PC were localized in potential survival niches characterized by CXCL12 as well as BAFF expression; however, PC survival niches in the brain need to be studied in more detail.

Only recently it became evident that also PC in the LP of the gut can be long-lived in mice and men (59, 60); again, this specific survival niche is less well defined. It is tempting to speculate that the cellular composition involved in PC survival niches in the GALT are as heterogeneous and dynamic as has been shown for stroma cells in the BM; but most likely, with different requirements due to the special location within the gut microenvironment.

PC in general have high metabolic needs to produce and secrete antibodies [reviewed in (2, 54, 61)]. Sitting in the villi of the LP, PC in the gut are exposed to bacterial antigens, nutrients, and varying oxygen concentrations in addition to soluble factors secreted by the surrounding cells. In an adaptation to this specific microenvironment, intestinal PC might have a distinct metabolic profile. For example, IgA-secreting PC exhibit higher expression of glycolysis-related metabolites than naïve B cells in PP or PC from the spleen (62). In addition, IgA ASC can utilize diet- and gut microbiota-derived short-chain fatty acids (SCFA) as one carbon source to maintain metabolism (63).

In addition to the defined metabolic compositions of PC niches, survival of PC can also be regulated by unique oxygenation profiles. Interestingly, switching to the IgA isotype is not affected by low oxygen conditions (64) but PC development seems to be increased under hypoxia (65). Inflammatory responses induced by environmental factors or intestinal dysbiosis might dramatically change oxygenation and the metabolic profile of the PC niches in the gut. However, little is known about how PC are maintained in those inflamed tissues and what kind of survival niches support their metabolic properties, function and survival.

## Modulating IgA ASC – Quo Vadis?

The here described findings indicate that the function of IgA ASC in local immune niches might be heavily influenced by the microenvironment but also by their education. The microbiota plays an essential role in the maturation and tolerance of the immune system, and hence also the differentiation of IgA ASC (66, 67). Autoimmune diseases are often associated with intestinal dysbiosis with specific classes of bacteria associated with certain disease (68, 69). Dysbiosis can be influenced by many aspects – environmental factors, antibiotics treatment, a genetic susceptibility of the host, but also aberrant IgA production. The cause or consequence of this dysbiosis and thus altered host-microbiota interaction remains to be studied in more detail, as modulation of the microbiome might positively influence systemic immunity and the outcome of disease (70).

In addition to the microbiome itself, recent studies have highlighted a strong influence of microbial metabolites in regulating host antibody responses. SCFA derived from the anaerobic fermentation of nondigestible polysaccharides such as dietary fibre counter inflammation and maintain gut homeostasis. Especially the SCFA butyrate and propionate have been described as either supporting or suppressing the generation of IgA PC (63, 71). These contradictory findings could be explained by the dose-dependent inhibitory effect of SCFA on PC differentiation, with low levels increasing, and higher levels restricting PC differentiation and CSR in TD and TI responses on an intestinal and systemic level (71). Besides impacting immune responses locally, SCFA also contribute to maintaining a healthy immune homeostasis systemically and prevent allergy and autoimmunity [reviewed in (72)].

The common notion is that IgA antibodies secreted during the here described auto-inflammatory settings have a different immunomodulatory role compared to IgA secreted under homeostatic conditions. The fact that mice only express one IgA subset, whereas human possess two IgA subclasses, IgA1 and IgA2, with different effector functions and glycosylation patterns, further adds to the layer of complexity. IgA1 is the prevalent form in human serum, but both subclasses are equally expressed in mucosal tissues. IgA2 is the more pro-inflammatory subset and which might increase during chronic inflammatory conditions (73). During inflammation, IgA (and IgG) antibodies differ by their glycosylation, with a reduced glycosylation profile often associated with disease severity (74). During IgAN for example, aberrantly circulatory IgA1 is elevated in patients (75–77). Antibody glycosylation also determines the outcome of the interaction of antibodies with their specific Fc receptors, thereby modulating inflammation (73, 78). It is tempting to speculate that the inflammatory intestinal PC niche determined by the composition of the microbiota and a distinct metabolic profile (e.g., availability of SCFA, oxygenation status) influences the glycosylation and effector function of IgA ASC. How pro- or anti-inflammatory effector functions and maybe migratory capacity of IgA-secreting PC are imprinted by the gut PC niche remains to be investigated. Similarly, other inflammatory niches such as in the kidney or the brain might influence effector functions of IgA ASC.

## Conclusions

**Figure 1:**
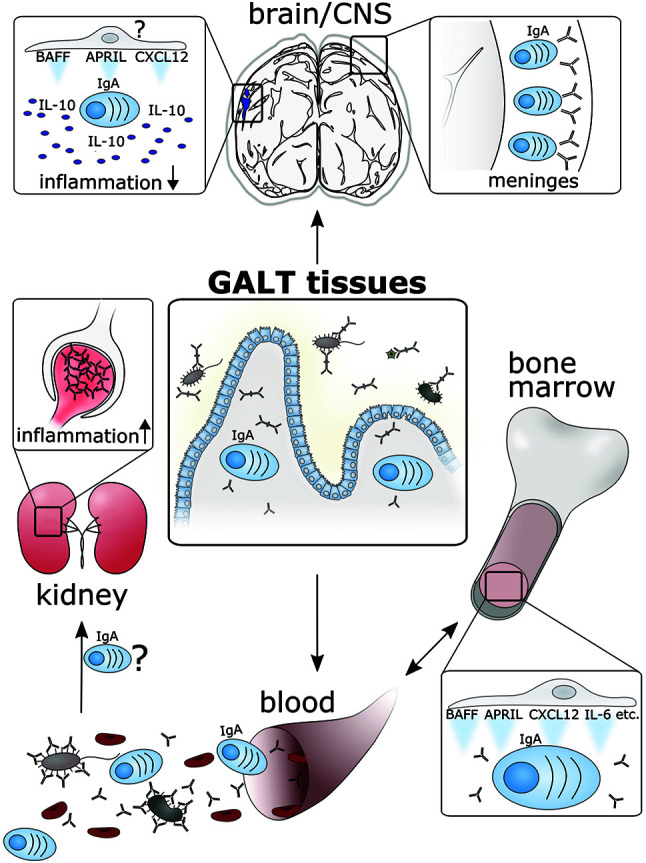
B cells differentiate into IgA ASC in the GALT due to the constant stimulation of the immune system by commensal bacteria, invading pathogens as well as food antigens. From there, gut-derived IgA ASC potentially travel through the blood to the PC niches in the BM. There, survival and function of PC can be supported by the secretion of exemplarily indicated survival factors. IgA ASC in the BM are thought to release monomeric IgA antibodies into the bloodstream directed against a variety of pathogens to counter sepsis in case of a microbial breach in the gut. Furthermore, IgA antibodies can be the cause for pathogenic immunoglobulin deposits in kidney glomeruli during autoinflammatory diseases. It is currently unknown, whether or not the infiltration of IgA ASC into the inflamed kidney further boosts disease through local antibody production. Finally, in the CNS, IgA PC protect the blood-brain barrier at the meninges from invading pathogens. Furthermore, gut-derived IgA PC can enter the CNS in inflammatory conditions like MS lesions, where they attenuate neuroinflammation in an IL-10 dependent manner. The brain might also provide factors needed for PC survival and hence constitute a novel PC niche. As more and more evidence points at the possibility of a systemic migration of gut-derived IgA ASC, further survival niches for those cells need to be considered. ASC, antibody secreting cell; GALT, gut associated lymphoid tissue; CNS, central nervous system; BM, bone marrow; PC, plasma cell; SLE, systemic lupus erythematosus; IgAN, IgA nephropathy; IL-10, interleukin-10.

We are only at the beginning to understand the impact of the interaction of gut-derived ASC with different immune niches on systemic immunity. In this Mini Review, we sum up recent evidence that IgA ASC potentially travel from the LP through the blood to the BM but can also be found in the kidney and CNS (**Figure 1**). During homeostasis, antibodies of gut-derived IgA ASC support microbial colonization, defend against systemic dissemination of harmful pathogens, prevent sepsis or pathogen infiltration of the CNS. In pathological settings, especially during autoimmune inflammation, IgA antibodies of gut-derived ASC seem to promote celiac disease or kidney malfunction, amongst others.

Besides antibody production, ASC of the IgM or IgG isotype have been shown to contribute to systemic immune responses through cytokine production (79). Gut-derived IgA ASC produce immunoregulatory IL-10 which seems to be beneficial in the inflamed CNS during mouse models of MS. On the other hand, IL-10 secretion by IgA ASC has been shown to suppress anti-tumour responses of CD8 T cells and hence is detrimental in both, prostate and liver tumour microenvironments (80, 81). However, in these studies, it seems that the local production of factors promoting IgA CSR (especially TGF-β) induces the differentiation and accumulation of IL-10 producing IgA ASC. The contribution of migrating IgA ASC in these settings is less clear.

As more and more evidence points at the possibility of a systemic migration of gut-derived IgA ASC, new survival niches for those cells need to be considered. Advances in single-cell and high-throughput techniques provide valuable tools; however, spatial information of ASC in their unique niche will also be necessary to understand these reciprocal interactions in their complexity. Novel imaging techniques such as volumetric imaging of tissue sections or whole mount organs (53, 56, 82) might help to detect rare IgA ASC and determine the specific microenvironment in order to better understand ASC function and local production of potentially harmful or protective IgA. A better understanding of the migration, function and survival of long-lived IgA PC in their specific niches is needed in order to modulate immune responses for therapeutic intervention.

## Author Contributions

SK: conceptualization, writing – original draft, writing – review and editing, funding acquisition. M-CG: writing – original draft. JH: writing – original draft, graphic. All authors contributed to the article and approved the submitted version.

## Funding

This work was supported by the Open-Access Publication Fonds of Technical University Munich (TUM), the German Research Foundation (DFG) grant Ke1737/2-1 (SJK), and the Else-Kröner-Fresenius-Stiftung grant 2019_A105 (SJK).

## Conflict of Interest

The authors declare that the research was conducted in the absence of any commercial or financial relationships that could be construed as a potential conflict of interest.

## References

[1] Nutt SL Hodgkin PD Tarlinton DM Corcoran LM. The generation of antibody-secreting plasma cells. Nat Rev Immunol (2015) 15(3):160–71. 10.1038/nri3795 25698678

[2] Schuh W Mielenz D Jack HM. Unraveling the mysteries of plasma cells. Adv Immunol (2020) 146:57–107. 10.1016/bs.ai.2020.01.002 32327153

[3] Tellier J Nutt SL. Plasma cells: The programming of an antibody-secreting machine. Eur J Immunol (2019) 49(1):30–7. 10.1002/eji.201847517 30273443

[4] Pabst O. New concepts in the generation and functions of IgA Nat Rev Immunol (2012) 12:821–32. 10.1038/nri3322 23103985

[5] Brandtzaeg P. Induction of secretory immunity and memory at mucosal surfaces. Vaccine (2007) 25(30):5467–84. 10.1016/j.vaccine.2006.12.001 17227687

[6] Moor K Diard M Sellin ME Felmy B Wotzka SY Toska A. High-avidity IgA protects the intestine by enchaining growing bacteria. Nature (2017) 544(7651):498–502. 10.1038/nature22058 28405025

[7] Pabst O Cerovic V Hornef M. Secretory IgA in the Coordination of Establishment and Maintenance of the Microbiota. Trends Immunol (2016) 37(5):287–96. 10.1016/j.it.2016.03.002 27066758

[8] Li C Lam E Perez-Shibayama C Ward LA Zhang J Lee D. Early-life programming of mesenteric lymph node stromal cell identity by the lymphotoxin pathway regulates adult mucosal immunity. Sci Immunol (2019) 4(42):1–17. 10.1126/sciimmunol.aax1027 31862865

[9] Reboldi A Arnon TI Rodda LB Atakilit A Sheppard D Cyster JG. IgA production requires B cell interaction with subepithelial dendritic cells in Peyer’s patches. Science (2016) 352(6287):aaf4822. 10.1126/science.aaf4822 27174992 PMC4890166

[10] Lecuyer E Rakotobe S Lengline-Garnier H Lebreton C Picard M Juste C. Segmented filamentous bacterium uses secondary and tertiary lymphoid tissues to induce gut IgA and specific T helper 17 cell responses. Immunity (2014) 40(4):608–20. 10.1016/j.immuni.2014.03.009 24745335

[11] Lochner M Ohnmacht C Presley L Bruhns P Si-Tahar M Sawa S. Microbiota-induced tertiary lymphoid tissues aggravate inflammatory disease in the absence of RORgamma t and LTi cells. J Exp Med (2011) 208(1):125–34. 10.1084/jem.20100052 PMC3023125 21173107

[12] Kruglov AA Grivennikov SI Kuprash DV Winsauer C Prepens S Seleznik GM. Nonredundant function of soluble LTalpha3 produced by innate lymphoid cells in intestinal homeostasis. Science (2013) 342(6163):1243–6. 10.1126/science.1243364 24311691

[13] Fagarasan S Kinoshita K Muramatsu M Ikuta K Honjo T. In situ class switching and differentiation to IgA-producing cells in the gut lamina propria. Nature (2001) 413(6856):639–43. 10.1038/35098100 11675788

[14] Cao AT Yao S Gong B Nurieva RI Elson CO Cong Y. Interleukin (IL)-21 promotes intestinal IgA response to microbiota. Mucosal Immunol (2015) 8(5):1072–82. 10.1038/mi.2014.134 PMC4501922 25586558

[15] Hirota K Turner JE Villa M Duarte JH Demengeot J Steinmetz OM. Plasticity of Th17 cells in Peyer’s patches is responsible for the induction of T cell-dependent IgA responses. Nat Immunol (2013) 14(4):372–9. 10.1038/ni.2552 PMC3672955 23475182

[16] Cazac BB Roes J. TGF-beta receptor controls B cell responsiveness and induction of IgA in vivo. Immunity (2000) 13(4):443–51. 10.1016/S1074-7613(00)00044-3 11070163

[17] Lee JM Jang YS Jin BR Kim SJ Kim HJ Kwon BE. Retinoic acid enhances lactoferrin-induced IgA responses by increasing betaglycan expression. Cell Mol Immunol (2016) 13(6):862–70. 10.1038/cmi.2015.73 PMC5101447 26277894

[18] Castigli E Wilson SA Scott S Dedeoglu F Xu S Lam KP. TACI and BAFF-R mediate isotype switching in B cells. J Exp Med (2005) 201(1):35–9. 10.1084/jem.20032000 PMC2212754 15630136

[19] Bunker JJ Flynn TM Koval JC Shaw DG Meisel M McDonald BD. Innate and Adaptive Humoral Responses Coat Distinct Commensal Bacteria with Immunoglobulin A. Immunity (2015) 43(3):541–53. 10.1016/j.immuni.2015.08.007 PMC4575282 26320660

[20] Macpherson AJ Gatto D Sainsbury E Harriman GR Hengartner H Zinkernagel RM. A primitive T cell-independent mechanism of intestinal mucosal IgA responses to commensal bacteria. Science (2000) 288(5474):2222–6. 10.1126/science.288.5474.2222 10864873

[21] Reynolds AE Kuraoka M Kelsoe G. Natural IgM is produced by CD5- plasma cells that occupy a distinct survival niche in bone marrow. J Immunol (2015) 194(1):231–42. 10.4049/jimmunol.1401203 PMC4272881 25429072

[22] Bunker JJ Erickson SA Flynn TM Henry C Koval JC Meisel M. Natural polyreactive IgA antibodies coat the intestinal microbiota. Science (2017) 358(6361):1–12. 10.1126/science.aan6619 PMC5790183 28971969

[23] Kabbert J Benckert J Rollenske T Hitch TCA Clavel T Cerovic V. High microbiota reactivity of adult human intestinal IgA requires somatic mutations. J Exp Med (2020) 217(11):1–14. 10.1084/jem.20200275 PMC7526496 32640466

[24] Lindner C Wahl B Fohse L Suerbaum S Macpherson AJ Prinz I. Age, microbiota, and T cells shape diverse individual IgA repertoires in the intestine. J Exp Med (2012) 209(2):365–77. 10.1084/jem.20111980 PMC3280880 22249449

[25] Pabst O Slack E. IgA and the intestinal microbiota: the importance of being specific. Mucosal Immunol (2020) 13(1):12–21. 10.1038/s41385-019-0227-4 31740744 PMC6914667

[26] Kunkel EJ Campbell JJ Haraldsen G Pan J Boisvert J Roberts AI. Lymphocyte CC chemokine receptor 9 and epithelial thymus-expressed chemokine (TECK) expression distinguish the small intestinal immune compartment: Epithelial expression of tissue-specific chemokines as an organizing principle in regional immunity. J Exp Med (2000) 192(5):761–8. 10.1084/jem.192.5.761 PMC2193265 10974041

[27] Kunkel EJ Kim CH Lazarus NH Vierra MA Soler D Bowman EP. CCR10 expression is a common feature of circulating and mucosal epithelial tissue IgA Ab-secreting cells. J Clin Invest (2003) 111(7):1001–10. 10.1172/JCI17244 PMC152588 12671049

[28] Hargreaves DC Hyman PL Lu TT Ngo VN Bidgol A Suzuki G. A coordinated change in chemokine responsiveness guides plasma cell movements. J Exp Med (2001) 194(1):45–56. 10.1084/jem.194.1.45 11435471 PMC2193440

[29] Hauser AE Debes GF Arce S Cassese G Hamann A Radbruch A. Chemotactic responsiveness toward ligands for CXCR3 and CXCR4 is regulated on plasma blasts during the time course of a memory immune response. J Immunol (2002) 169(3):1277–82. 10.4049/jimmunol.169.3.1277 12133949

[30] Mei HE Yoshida T Sime W Hiepe F Thiele K Manz RA. Blood-borne human plasma cells in steady state are derived from mucosal immune responses. Blood (2009) 113(11):2461–9. 10.1182/blood-2008-04-153544 18987362

[31] Iversen R Snir O Stensland M Kroll JE Steinsbo O Korponay-Szabo IR. Strong Clonal Relatedness between Serum and Gut IgA despite Different Plasma Cell Origins. Cell Rep (2017) 20(10):2357–67. 10.1016/j.celrep.2017.08.036 PMC5603730 28877470

[32] Mei HE Wirries I Frolich D Brisslert M Giesecke C Grun JR. A unique population of IgG-expressing plasma cells lacking CD19 is enriched in human bone marrow. Blood (2015) 125(11):1739–48. 10.1182/blood-2014-02-555169 25573986

[33] Bemark M Hazanov H Stromberg A Komban R Holmqvist J Koster S. Limited clonal relatedness between gut IgA plasma cells and memory B cells after oral immunization. Nat Commun (2016) 7:12698. 10.1038/ncomms12698 27596266 PMC5025876

[34] Lemke A Kraft M Roth K Riedel R Lammerding D Hauser AE. Long-lived plasma cells are generated in mucosal immune responses and contribute to the bone marrow plasma cell pool in mice. Mucosal Immunol (2016) 9(1):83–97. 10.1038/mi.2015.38 25943272

[35] Pracht K Meinzinger J Daum P Schulz SR Reimer D Hauke M. A new staining protocol for detection of murine antibody-secreting plasma cell subsets by flow cytometry. Eur J Immunol (2017) 47(8):1389–92. 10.1002/eji.201747019 28608550

[36] Rojas OL Probstel AK Porfilio EA Wang AA Charabati M Sun T. Recirculating Intestinal IgA-Producing Cells Regulate Neuroinflammation via IL-10. Cell (2019) 177(2):492–3. 10.1016/j.cell.2019.03.037 30951673

[37] Wilmore JR Gaudette BT Gomez Atria D Hashemi T Jones DD Gardner CA. Commensal Microbes Induce Serum IgA Responses that Protect against Polymicrobial Sepsis. Cell Host Microbe (2018) 23(3):302–11.e3. 10.1016/j.chom.2018.01.005 29478774 PMC6350773

[38] Wyatt RJ Julian BA. IgA nephropathy. N Engl J Med (2013) 368(25):2402–14. 10.1056/NEJMra1206793 23782179

[39] Barratt J Rovin BH Cattran D Floege J Lafayette R Tesar V. Why Target the Gut to Treat IgA Nephropathy? Kidney Int Rep (2020) 5(10):1620–4. 10.1016/j.ekir.2020.08.009 PMC7569689 33102954

[40] Floege J Feehally J. The mucosa-kidney axis in IgA nephropathy. Nat Rev Nephrol (2016) 12(3):147–56. 10.1038/nrneph.2015.208 26714580

[41] McCarthy DD Kujawa J Wilson C Papandile A Poreci U Porfilio EA. Mice overexpressing BAFF develop a commensal flora-dependent, IgA-associated nephropathy. J Clin Invest (2011) 121(10):3991–4002. 10.1172/JCI45563 21881212 PMC3195458

[42] Jacobi AM Odendahl M Reiter K Bruns A Burmester GR Radbruch A. Correlation between circulating CD27high plasma cells and disease activity in patients with systemic lupus erythematosus. Arthritis Rheum (2003) 48(5):1332–42. 10.1002/art.10949 12746906

[43] Odendahl M Jacobi A Hansen A Feist E Hiepe F Burmester GR. Disturbed peripheral B lymphocyte homeostasis in systemic lupus erythematosus. J Immunol (2000) 165(10):5970–9. 10.4049/jimmunol.165.10.5970 11067960

[44] Cassese G Lindenau S de Boer B Arce S Hauser A Riemekasten G. Inflamed kidneys of NZB / W mice are a major site for the homeostasis of plasma cells. Eur J Immunol (2001) 31(9):2726–32. 10.1002/1521-4141(200109)31:9<2726::AID-IMMU2726>3.0.CO;2-H 11536171

[45] Starke C Frey S Wellmann U Urbonaviciute V Herrmann M Amann K. High frequency of autoantibody-secreting cells and long-lived plasma cells within inflamed kidneys of NZB/W F1 lupus mice. Eur J Immunol (2011) 41(7):2107–12. 10.1002/eji.201041315 21484784

[46] Espeli M Bokers S Giannico G Dickinson HA Bardsley V Fogo AB. Local renal autoantibody production in lupus nephritis. J Am Soc Nephrol (2011) 22(2):296–305. 10.1681/ASN.2010050515 21088295 PMC3029902

[47] Mei HE Hahne S Redlin A Hoyer BF Wu K Baganz L. Plasmablasts With a Mucosal Phenotype Contribute to Plasmacytosis in Systemic Lupus Erythematosus. Arthritis Rheumatol (2017) 69(10):2018–28. 10.1002/art.40181 28622453

[48] Hepworth MR Greenhalgh AD Cook PC. B cells on the brain: meningeal IgA and a novel gut-brain firewall. Immunol Cell Biol (2021) 99(1):17–20. 10.1111/imcb.12412 33107992

[49] Fitzpatrick Z Frazer G Ferro A Clare S Bouladoux N Ferdinand J. Gut-educated IgA plasma cells defend the meningeal venous sinuses. Nature (2020) 587(7834):472–6. 10.1038/s41586-020-2886-4 PMC7748383 33149302

[50] Probstel AK Zhou X Baumann R Wischnewski S Kutza M Rojas OL. Gut microbiota-specific IgA(+) B cells traffic to the CNS in active multiple sclerosis. Sci Immunol (2020) 5(53):1–13. 10.1126/sciimmunol.abc7191 PMC8043673 33219152

[51] Manz RA Thiel A Radbruch A. Lifetime of plasma cells in the bone marrow. Nature (1997) 388(6638):133–4. 10.1038/40540 9217150

[52] Amanna IJ Carlson NE Slifka MK. Duration of humoral immunity to common viral and vaccine antigens. N Engl J Med (2007) 357(19):1903–15. 10.1056/NEJMoa066092 17989383

[53] Lindquist RL Niesner RA Hauser AE. In the Right Place, at the Right Time: Spatiotemporal Conditions Determining Plasma Cell Survival and Function. Front Immunol (2019) 10:788. 10.3389/fimmu.2019.00788 31068930 PMC6491733

[54] Lightman SM Utley A Lee KP. Survival of Long-Lived Plasma Cells (LLPC): Piecing Together the Puzzle. Front Immunol (2019) 10:965. 10.3389/fimmu.2019.00965 31130955 PMC6510054

[55] Addo RK Heinrich F Heinz GA Schulz D Sercan-Alp O Lehmann K. Single-cell transcriptomes of murine bone marrow stromal cells reveal niche-associated heterogeneity. Eur J Immunol (2019) 49(9):1372–9. 10.1002/eji.201848053 PMC6771914 31149730

[56] Holzwarth K Kohler R Philipsen L Tokoyoda K Ladyhina V Wahlby C. Multiplexed fluorescence microscopy reveals heterogeneity among stromal cells in mouse bone marrow sections. Cytometry A (2018) 93(9):876–88. 10.1002/cyto.a.23526 30107096

[57] Nguyen DC Garimalla S Xiao H Kyu S Albizua I Galipeau J. Factors of the bone marrow microniche that support human plasma cell survival and immunoglobulin secretion. Nat Commun (2018) 9(1):3698. 10.1038/s41467-018-05853-7 30209264 PMC6135805

[58] Pollok K Mothes R Ulbricht C Liebheit A Gerken JD Uhlmann S. The chronically inflamed central nervous system provides niches for long-lived plasma cells. Acta Neuropathol Commun (2017) 5(1):88. 10.1186/s40478-017-0487-8 29178933 PMC5702095

[59] Hapfelmeier S Lawson MA Slack E Kirundi JK Stoel M Heikenwalder M. Reversible microbial colonization of germ-free mice reveals the dynamics of IgA immune responses. Science (2010) 328(5986):1705–9. 10.1126/science.1188454 PMC3923373 20576892

[60] Landsverk OJ Snir O Casado RB Richter L Mold JE Reu P. Antibody-secreting plasma cells persist for decades in human intestine. J Exp Med (2017) 214(2):309–17. 10.1084/jem.20161590 PMC5294861 28104812

[61] Jellusova J Rickert RC. A Brake for B Cell Proliferation: Appropriate responses to metabolic stress are crucial to maintain B cell viability and prevent malignant outgrowth. Bioessays (2017) 39(11):1–9. 10.1002/bies.201700079 28961325

[62] Kunisawa J Sugiura Y Wake T Nagatake T Suzuki H Nagasawa R. Mode of Bioenergetic Metabolism during B Cell Differentiation in the Intestine Determines the Distinct Requirement for Vitamin B1. Cell Rep (2015) 13(1):122–31. 10.1016/j.celrep.2015.08.063 26411688

[63] Kim M Qie Y Park J Kim CH. Gut Microbial Metabolites Fuel Host Antibody Responses. Cell Host Microbe (2016) 20(2):202–14. 10.1016/j.chom.2016.07.001 PMC4982788 27476413

[64] Cho SH Raybuck AL Stengel K Wei M Beck TC Volanakis E. Germinal centre hypoxia and regulation of antibody qualities by a hypoxia response system. Nature (2016) 537(7619):234–8. 10.1038/nature19334 PMC5161594 27501247

[65] Abbott RK Thayer M Labuda J Silva M Philbrook P Cain DW. Germinal Center Hypoxia Potentiates Immunoglobulin Class Switch Recombination. J Immunol (2016) 197(10):4014–20. 10.4049/jimmunol.1601401 PMC5123804 27798169

[66] Macpherson AJ Yilmaz B Limenitakis JP Ganal-Vonarburg SC. IgA Function in Relation to the Intestinal Microbiota. Annu Rev Immunol (2018) 36:359–81. 10.1146/annurev-immunol-042617-053238 29400985

[67] Zheng D Liwinski T Elinav E. Interaction between microbiota and immunity in health and disease. Cell Res (2020) 30(6):492–506. 10.1038/s41422-020-0332-7 32433595 PMC7264227

[68] Gianchecchi E Fierabracci A. Recent Advances on Microbiota Involvement in the Pathogenesis of Autoimmunity. Int J Mol Sci (2019) 20(2):1–28. 10.3390/ijms20020283 PMC6359510 30642013

[69] Zhang X Chen BD Zhao LD Li H. The Gut Microbiota: Emerging Evidence in Autoimmune Diseases. Trends Mol Med (2020) 26(9):862–73. 10.1016/j.molmed.2020.04.001 32402849

[70] Brown J Robusto B Morel L. Intestinal Dysbiosis and Tryptophan Metabolism in Autoimmunity. Front Immunol (2020) 11:1741. 10.3389/fimmu.2020.01741 32849620 PMC7417361

[71] Sanchez HN Moroney JB Gan H Shen T Im JL Li T. B cell-intrinsic epigenetic modulation of antibody responses by dietary fiber-derived short-chain fatty acids. Nat Commun (2020) 11(1):60. 10.1038/s41467-019-13603-6 31896754 PMC6940392

[72] Veldhoen M Ferreira C. Influence of nutrient-derived metabolites on lymphocyte immunity. Nat Med (2015) 21(7):709–18. 10.1038/nm.3894 26121194

[73] Steffen U Koeleman CA Sokolova MV Bang H Kleyer A Rech J. IgA subclasses have different effector functions associated with distinct glycosylation profiles. Nat Commun (2020) 11(1):120. 10.1038/s41467-019-13992-8 31913287 PMC6949214

[74] Reily C Stewart TJ Renfrow MB Novak J. Glycosylation in health and disease. Nat Rev Nephrol (2019) 15(6):346–66. 10.1038/s41581-019-0129-4 PMC6590709 30858582

[75] Novak J Barratt J Julian BA Renfrow MB. Aberrant Glycosylation of the IgA1 Molecule in IgA Nephropathy. Semin Nephrol (2018) 38(5):461–76. 10.1016/j.semnephrol.2018.05.016 PMC7170174 30177018

[76] Allen AC Bailey EM Brenchley PE Buck KS Barratt J Feehally J. Mesangial IgA1 in IgA nephropathy exhibits aberrant O-glycosylation: observations in three patients. Kidney Int (2001) 60(3):969–73. 10.1046/j.1523-1755.2001.060003969.x 11532091

[77] Hiki Y Odani H Takahashi M Yasuda Y Nishimoto A Iwase H. Mass spectrometry proves under-O-glycosylation of glomerular IgA1 in IgA nephropathy. Kidney Int (2001) 59(3):1077–85. 10.1046/j.1523-1755.2001.00591.x 11231363

[78] Nimmerjahn F Ravetch JV. Fcgamma receptors as regulators of immune responses. Nat Rev Immunol (2008) 8(1):34–47. 10.1038/nri2206 18064051

[79] Wang AA Gommerman JL Rojas OL. Plasma Cells: From Cytokine Production to Regulation in Experimental Autoimmune Encephalomyelitis. J Mol Biol (2021) 433(1):166655. 10.1016/j.jmb.2020.09.014 32976908

[80] Shalapour S Font-Burgada J Di Caro G Zhong Z Sanchez-Lopez E Dhar D. Immunosuppressive plasma cells impede T-cell-dependent immunogenic chemotherapy. Nature (2015) 521(7550):94–8. 10.1038/nature14395 PMC4501632 25924065

[81] Shalapour S Lin XJ Bastian IN Brain J Burt AD Aksenov AA. Inflammation-induced IgA+ cells dismantle anti-liver cancer immunity. Nature (2017) 551(7680):340–5. 10.1038/nature24302 PMC5884449 29144460

[82] Hofmann J Gadjalova I Mishra R Ruland J Keppler SJ. Efficient Tissue Clearing and Multi-Organ Volumetric Imaging Enable Quantitative Visualization of Sparse Immune Cell Populations During Inflammation. Front Immunol (2020) 11:599495. 10.3389/fimmu.2020.599495 33569052 PMC7869862

